# Evaluation of Hemoadsorption Capacity of the CytoSorb Adsorber for Toxic Doses of Diclofenac and Meloxicam in Porcine Plasma

**DOI:** 10.1002/jca.70130

**Published:** 2026-05-01

**Authors:** Bettina Giani, René Dörfelt, Katrin Hartmann, Florian Sänger

**Affiliations:** ^1^ LMU Small Animal Clinic, Center for Clinical Veterinary Medicine, Faculty of Veterinary Medicine Ludwig‐Maximilians‐Universität München Munich Germany

**Keywords:** drug elimination, extracorporeal blood purification, extracorporeal circuit, hemoadsorption, non‐steroidal anti‐inflammatory drugs

## Abstract

The objective of this in vitro experimental was to determine the utility of hemoadsorption (HA) using the CytoSorb adsorber to remove meloxicam and diclofenac from porcine plasma. For this purpose 12 1‐L aliquots of porcine plasma, 6 units were spiked with 293 μg/mL (262–320 μg/mL) diclofenac and 6 with 42.8 μg/mL (41.4–44.7 μg/mL) meloxicam, respectively. Each unit was processed in a circular setup with the CytoSorb adsorber using the PuriFi HA platform until a recirculated volume of 20 L was reached. Pre‐ and post‐adsorber samples were obtained at seven time points. Percentage reduction, clearance, and change from baseline were calculated. Drug concentrations were compared using Friedmann test with post hoc Dunn's multiple comparison test and Wilcoxon matched pairs rank sum test. *p* < 0.05 were considered significant. Diclofenac was reduced by a median of 242 mg (204–262 mg; 82.1%; 77.0%–83.6%). Total clearance was 40.5 mL/min (33.2–46.2 mL/min). Clearance at a plasma flow of 100 mL/min decreased from 39.9 mL/min (25.3–46.6 mL/min) at 0.2 L to 6.4 mL/min (−3.8 to 14.8 mL/min) at 20 L. Median reduction of meloxicam was 40.4 mg (38.6–42.2 mg; 94.4%; 93.2%–94.6%); total clearance was 94.38 mL/min (93.2–94.6 mL/min). Clearance at a plasma flow of 100 mL/min decreased from 65.3 mL/min (56.7–71.7 mL/min) at 0.2 L to 3.6 mL/min (−13.0 to 4.2 mL/min) at 20 L. HA using the CytoSorb adsorber successfully removed meloxicam and diclofenac from porcine plasma.

## 1 Introduction

Overdose and intoxication with non‐steroidal anti‐inflammatory drugs (NSAID) is a common emergency that can result from low‐threshold availability, unintended ingestion by adults or children, or administration of overdosed or inappropriate medication [1]. NSAIDs can, dose‐dependently or idiosyncratically, lead to serious organ damage, such as renal, hepatic, or gastrointestinal injury [2, 3, 4].

Meloxicam and diclofenac are two NSAID representatives. Diclofenac (2‐[2‐(2,6‐dichloroanilino)phenyl]acetic acid) is a phenylacetic acid derivative and inhibitor of prostaglandin synthesis via cyclooxygenase‐2 (COX‐2) inhibition and is categorized by a low molecular weight (MW) of 296.1 g/mol [5]. The volume of distribution (VD) is 0.1–0.2 L/kg, protein binding is > 99.7%, and its elimination half‐life is 1–2 h. In vivo, diclofenac is hydroxylated and conjugated in the liver and undergoes rapid elimination. It is excreted predominantly via the kidney, but also in parts in bile and feces. The therapeutic dose ranges between 50 and 75 mg per individuum two to three times daily. The reported maximum therapeutic dose for adults is 150 mg/day in divided doses [5, 6, 7, 8].

The enolcarboxamide meloxicam (4‐hydroxy‐2‐methyl‐*N*‐(5‐methyl‐2‐thiazolyl)‐2H‐1,2‐benzothiazine‐3‐carboxamide‐1,1‐dioxide) is an inhibitor of prostaglandin synthesis, mediated by a preferential, but not exclusive, inhibition of COX‐2. As most NSAIDs, meloxicam is extensively protein‐bound (> 99%). It has a VD of 0.3 L/kg, a MW of 351.4 g/mol and a plasma half‐life of 20 h [9]. In vivo, orally administered meloxicam undergoes extensive hepatic metabolism and enterohepatic recirculation. The inactive metabolites are excreted via urine and feces [10, 11]. The lowest therapeutic dose is 7.5 mg/day; the maximum daily dose is 15 mg/day in adults [6, 8].

Traditional management of NSAID intoxication is composed of six phases: (1) stabilization, (2) laboratory assessment, (3) decontamination of gastrointestinal tract, skin or eyes, (4) antidote administration, where available, (5) elimination of the drug, and (6) observation and development of the follow‐up treatment plan [12]. Extracorporeal blood purification techniques, such as hemodialysis (HD), hemoadsorption (HA), and therapeutic plasma exchange (TPE), can be useful to enhance elimination of a substance by removing a large proportion of the drug from the blood before further organ damage occurs [13, 14, 15]. Large solutes or substances with high protein binding, such as most NSAIDs, are not readily removed across a hollow fiber membrane as used in HD [15]. For those cases, TPE or HA are the methods of choice [14]. HA might be a viable alternative to TPE for extracorporeal treatment, sparing the need for and potential risks of donor plasma, which is required for TPE.

The mechanism of solute adsorption in HA is based on the principle of mass separation by a solid agent. The binding of substances to the sorbent is mediated by weak ionic bonds, Van der Waals forces, and strong hydrophobic bonds [16]. Historically, an activated carbon filter was used for toxin removal via HA. Issues regarding biocompatibility, causing adverse effects such as thrombocytopenia, leukopenia, hypocalcemia and hypoglycemia, led to a decrease in the use of HA [16, 17]. This changed with the introduction of the CytoSorb hemoadsorber system in 2011, with improvements in bio‐ and hemocompatibility [16]. Following emergency authorization by the FDA in 2020, a marked increase in the use of the CytoSorb adsorber was observed, accompanied by a substantial rise in the associated scientific literature [16, 18, 19, 20, 21]. The CytoSorb adsorber primarily binds low to medium MW substances up to a MW of approximately 60 kDa, such as pro‐ and anti‐inflammatory cytokines, bilirubin or myoglobin, and to a smaller extent plasma proteins > 100 kDa [22]. Besides its use to remove excess cytokines from the body in patients with sepsis or systemic inflammation to ameliorate cytokine storm, the adsorber was found to bind a broad range of other substrates, such as hormones, various antibiotics, antithrombotics and immunosuppressives [19, 23, 24]. Cumulative evidence supports the use of the CytoSorb HA technology in the treatment of acute drug overdose of phenobarbital, 3,4‐methylenedioxymethamphetamine (MDMA), tricyclic antidepressants, and clozapine [25, 26, 27, 28, 29]. As the CytoSorb adsorber binds substances depending on their concentration in blood, this HA device is particularly advantageous for the removal of high doses of toxic substances. The whole‐blood cartridge of the adsorber contains highly porous bio‐ and hemocompatible polystyrene divinylbenzene beads, resulting in extensive binding capacity with a total surface area of 45 000 m^2^ per cartridge [16]. The CytoSorb adsorber has a strong safety profile. The adsorber itself is easy to store without the need for special storage conditions [16]. Based on their chemical and structural properties, diclofenac and meloxicam are deemed suitable substrates for the adsorption by the CytoSorb adsorber [13].

This study aims to determine the in vitro effect of HA using the CytoSorb adsorber on porcine plasma spiked with 10‐fold maximum therapeutic doses of diclofenac and meloxicam regarding its total removal capacity and changes in clearance over time.

It was hypothesized that the CytoSorb adsorber would be highly effective in binding the respective substances and that treatment of the plasma units with HA would significantly reduce plasma concentrations of both drugs in an in vitro setting and that clearance would be highest in the initial phase of the experiment and decrease over time.

## 2 Materials and Methods

### 2.1 Ethical Approval

This study was approved by the ethical committee of the Center of Clinical Veterinary Medicine of the Ludwig‐Maximilians‐University Munich (number 430‐21‐11‐24).

### 2.2 Study Design

For this in vitro study, porcine blood was collected in a slaughterhouse during slaughtering process. Blood was anticoagulated with 10 I.U./mL heparin (Heparin‐Natrium 25 000 I.E./5 mL Inj. Lsg., B. Braun, Melsungen, Germany). The whole blood was divided into 500 mL portions and centrifuged at 4°C at 2500*g* for 15 min in a large‐volume, temperature‐controlled centrifuge (Hettich ROTANTA 460 R, Andreas Hettich GmbH, Tuttlingen, Germany) to separate plasma from cells. The resultant plasma was divided into aliquots of 1000 mL each and stored at 4°C for a maximum of 3 days before use.

For study purposes, the in vitro circuit was downscaled in a 1:3.5 ratio. A 10‐fold therapeutic dose for a 70 kg individuum was extrapolated to 1 L of plasma. The calculated amount for the respective NSAID was added to the plasma unit immediately before the start of the experiment. The experiment was carried out six times for each substance, using a new plasma unit and adsorber for each run, analyzing only one substance per plasma unit.

For diclofenac, a dose of 1500 mg resulted in a calculated starting concentration of 428 μg/mL diclofenac (Diclofenac‐ratiopharm 75 mg/2 mL, Ratiopharm GmbH, Ulm, Germany). For meloxicam, a dose of 150 mg resulted in a starting concentration of 43 μg/mL meloxicam (Metacam 20 mg/mL Injektionslösung für Pferde und Schweine, Böhringer Ingelheim Vetmedica GmbH, Ingelheim am Rhein, Germany). Following the injection of the respective substance, each unit was mixed by turning it upside down 10 times to ensure thorough mixing. Subsequently, every plasma unit was treated with HA using a CytoSorb adsorber. The experiments were carried out six times for each substance.

### 2.3 Experimental Setup

**FIGURE 1:**
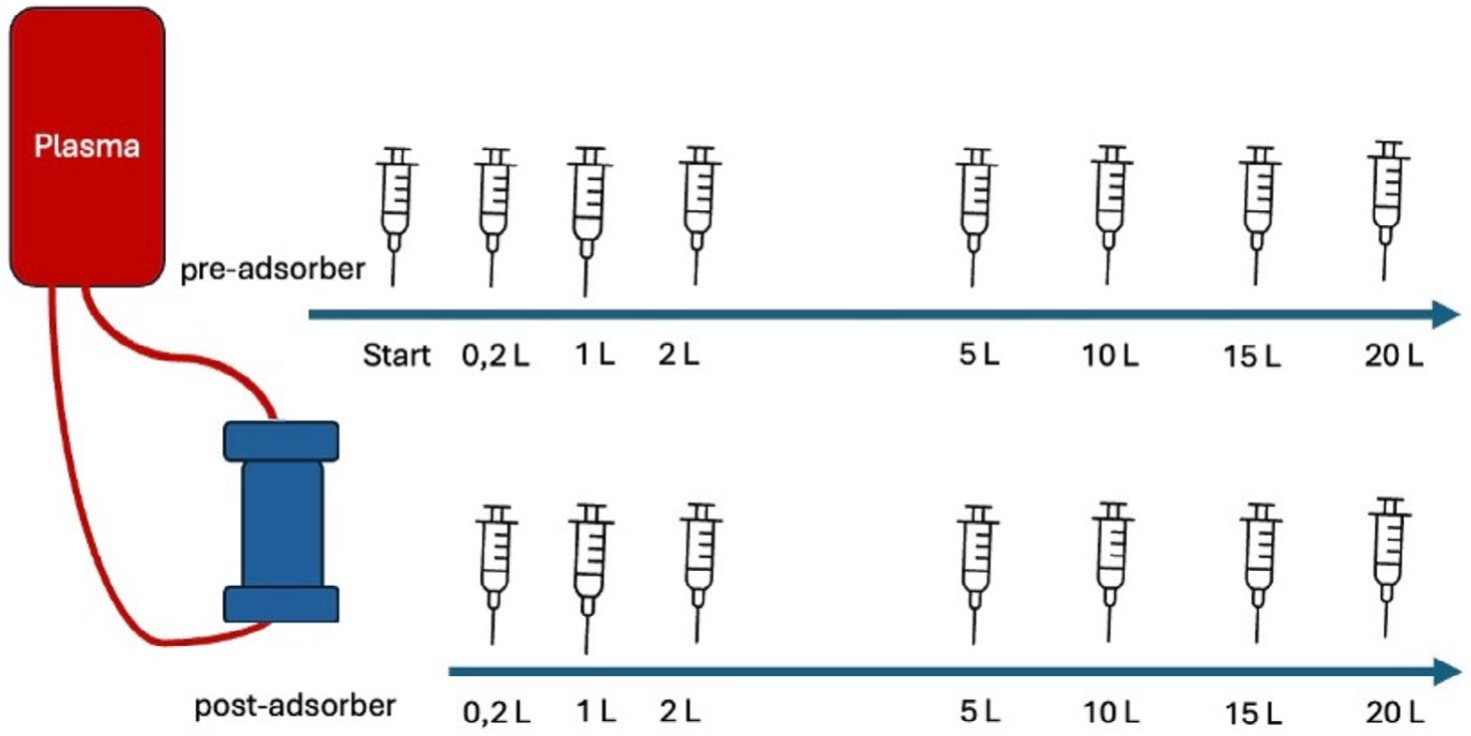
Experimental setup: Schematic timeline of sampling time‐points for determination of diclofenac and meloxicam concentrations in porcine plasma pre‐ and post‐adsorber during hemoadsorption at a plasma flow rate of 100 mL/min.

The plasma was circulated in an in vitro HA circuit using a standalone HA machine (PuriFi, CytoSorbents Europe GmbH, Berlin, Germany). The machine was prepared following the manufacturer's instructions using a standard HA tubing set (M90444 PuriFi tubing set, CytoSorbents Europe GmbH, Berlin, Germany) consisting of an access and a return line to be connected to the CytoSorb HA column containing 300 mL of sorbent material [30]. After preparation, the in vitro circuit was primed with isotonic saline (NaCl 0.9%, B. Braun Vet Care AG, Melsungen, Germany) before the adsorber connection. After priming the whole circuit with isotonic saline, the plasma unit was connected to the system in a serial patient connection manner. This mode of connection allowed to minimize additional dilution of the plasma, as the system was filled with plasma before connection of the return line to the plasma bag. After discarding 200 mL of saline, the return line was connected to the plasma reservoir and blood flow was established at a set recirculation rate of 100 mL/min, until a total plasma volume of 20 L was processed in a circulatory setup (Figure 1). For anticoagulation, heparin, diluted with isotonic saline to a concentration of 2000 I.U./ml, was used at a rate of 1 mL/h (2000 I.U./h).

One plasma sample was collected before starting the procedure to determine a baseline concentration of the respective NSAID in each unit of plasma. Pre‐ and post‐adsorber samples were collected at the respective sampling ports at seven time points (after 0.2, 1, 2, 5, 10, 15, and 20 L of processed plasma).

### 2.4 Laboratory Analysis

Samples were stored at −30°C for a maximum of 2 weeks and shipped to an external laboratory (MVZ—Dr. Eberhard & Partner Dortmund, Dortmund, Germany) for batch analysis. Substance concentrations were determined using liquid chromatography with tandem mass spectrometry (LC–MS/MS) technique. The concentrations of diclofenac and meloxicam were analyzed individually for each compound at each sampling time point. For meloxicam and diclofenac, the lower level of quantification (LLOQ) was 0.01 and 0.2 μg/mL with a linear range of 0.1–5.0 μg/mL and 0.05–2.5 μg/mL, respectively.

As the analytical method had not been validated for porcine plasma, spike‐and‐recovery experiments with porcine plasma were performed for both analytes. Percent recovery was calculated using the following formula: ((*C*_spiked_ − *C*_unspiked_)/*C*_added_) × 100, where *C*_spiked_ is the concentration in plasma spiked with a known amount of the compound, *C*_unspiked_ is the measured concentration of the respective substance in unspiked plasma, and *C*_added_ refers to the amount of substance added to unspiked plasma.

### 2.5 Statistical Methods

Statistical analysis was performed using commercially available software (Prism 5 for Windows; GraphPad Software, San Diego, CA). NSAID concentrations were reported as median and range (minimum–maximum). Total drug removal (change from baseline) and total clearance were calculated for each substance after processing of 20 L of plasma. Change in clearance over time and adsorber clearance were calculated for each sampling point. Values at one sample collection point (pre‐ or post‐adsorber) were compared using the Friedman test and post hoc Dunn's multiple comparison test. Concentration differences pre‐ and post‐adsorber were analyzed using the Wilcoxon matched‐pairs‐rank‐sum test. The total drug reduction, percentage drug reduction and total clearance after 20 L of processed plasma is reported as median (minimum—maximum) and 95% confidence interval (95% CI). *p* < 0.05 was considered statistically significant.

Adsorber clearance (CL_ad_) was calculated based on the extraction ratio across the cartridge and the plasma flow rate, according to the following formula:

$${\mathrm{CL}}_{\mathrm{ad}}=Q_{p}\times E$$

where *Q*_p_ is the plasma flow rate (mL/min), and *E* is the extraction ratio, defined as follows:

$$E=\left(C_{\text{in}}—C_{\text{out}}\right)/C_{\text{in}}$$

with *C*_in_ and *C*_out_ representing pre‐ and post‐adsorber substance concentrations, respectively.

Cumulative substance removal over time was assessed using mass balance calculations derived from concentration changes in the reservoir:

$$M_{\mathrm{cum}}=V\times \left(C_{\left(t0\right)}–C_{\left(t20\right)}\right)$$

where *V* is the total plasma volume in the circuit, *C*_(*t*0)_ is the initial drug concentration, and *C*_(*t*20)_ is the concentration at time t = 20 L of processed plasma.

The percentage reduction for each substance was calculated as follows:

$$\text{Percentage reduction}=\left(\left(c_{\text{in}}\;t=0\right)–\left(c_{\text{in}}\;t=20\right)\right)/\left(c_{\text{in}}\;t=0\right)\times 100\%$$

with the concentration of the respective substance at the beginning (0 L of processed plasma) and the end (20 L) of the experiment.

## 3 Results

Spike‐and‐recovery experiments were performed for both substances and results are reported as median and range. For diclofenac, a median recovery of 80% (77.6%–82.0%) and 79% (75.7%–82.0%) was found in porcine and human plasma, respectively. A higher recovery rate was demonstrated for meloxicam with 95.7% (88.5%–96.4%) and 93% (80.5%–95.3%) in porcine and human plasma, respectively.

### 3.1 Diclofenac

**FIGURE 2:**
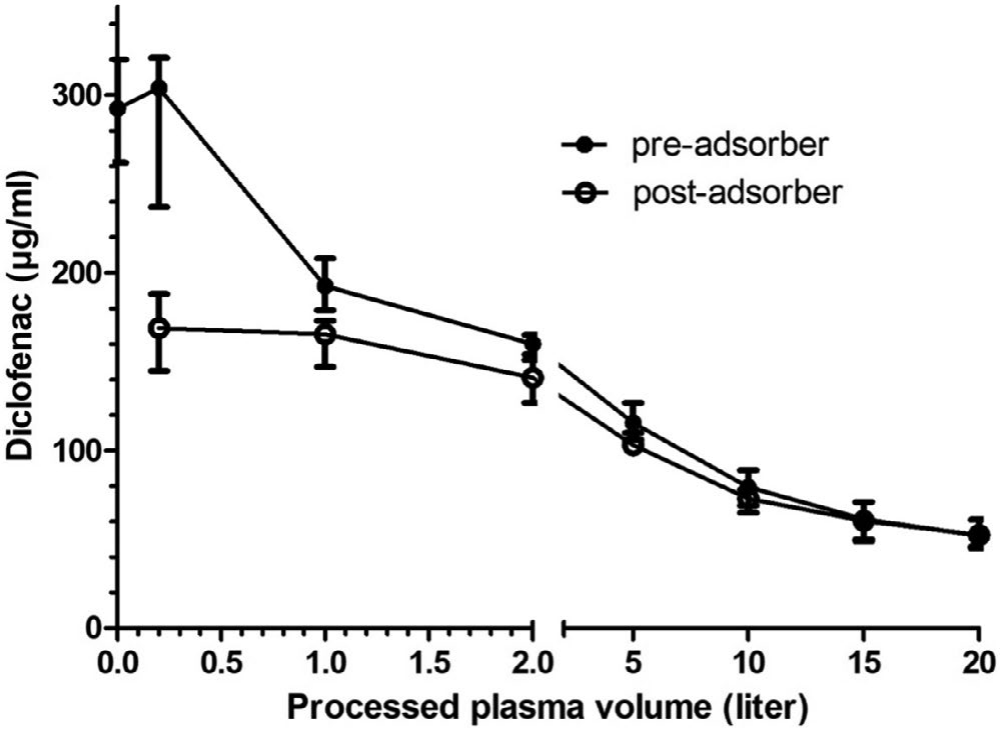
Median and range (minimum–maximum) of pre‐ and post‐adsorber concentrations of diclofenac in 1 L of porcine plasma spiked with 293 mg diclofenac during six test runs. Minimum and maximum concentrations (μg/mL) are represented by the lower and upper bar, respectively; median concentration is represented by a dot (pre‐adsorber) and a circle (post‐adsorber). Hemoadsorption was performed at a plasma flow rate of 100 mL/min using the CytoSorb adsorber.

The median recovery rate for diclofenac in porcine plasma was 68.5% with an intended concentration of 428 μg/mL and a reached concentration at the starting point (0 L of processed plasma) of 293 μg/mL (262–320 μg/mL). The pre‐adsorber diclofenac concentration of 293 μg/mL (262–320 μg/mL) decreased significantly between the time points 0 and 15 and 20 L, as well as between 0.2 and 10, 15, 20 L and 1 L and 20 L (*p* < 0.001, Figure 2, Table 1). The post‐adsorber diclofenac concentration of 169 μg/mL (145–188 μg/mL) at 0.2 L decreased significantly between the time points 0.2 and 15 and 20 L, as well as between 1 L and 15 and 20 L, and 2 and 20 L (*p* < 0.001, Figure 2, Table 1).

**TABLE 1:** Diclofenac concentration in 1 L of porcine plasma spiked with 293 mg diclofenac during hemoadsorption using the CytoSorb adsorber.

Processed plasma, volume (L)	*n*	0	0.2	1	2	5	10	15	20	*p* (Friedman test)
Diclofenac pre‐adsorber (μg/mL)	6	293 (262–320)	304 (237–321)	193 (179–208)	160 (154–165)	116 (106–127)	80 (69–89)^b^	62 (49–66)^a^, ^b^	53 (45–61)^a^, ^b^, ^c^	**< 0.001**
Dicolfenac post‐adsorber (μg/mL)	6		169 (145–188)	166 (147–173)	141 (127–151)	103 (99–110)	73 (65–78)	61 (50–71)^b^, ^c^	53 (46–54)^b^, ^c^, ^d^	**< 0.001**
*p* (Wilcoxon matched pairs signed rank test)	6		**0.031**	**0.036**	**0.031**	**0.036**	**0.031**	1.000	0.461	

*Note:* Samples were collected before (pre) and after (post) the adsorber after processing 0–20 L of plasma. Concentrations are reported as median and range (minimum–maximum). Diclofenac values at each sample collection point (pre‐adsorber and post‐adsorber) are analyzed using the Friedman test and post hoc Dunn's multiple comparison test. Concentration differences before and after the adsorber were analyzed using the Wilcoxon matched‐pairs signed‐rank test. The bold values indicate statistical significance based on *p* < 0.05.

Abbreviation: *n* = number of test runs.

^a^ Different to 0.

^b^ Differnt to 0.2 L.

^c^ Different to 1 L.

^d^ Different to 2 L.

The total diclofenac reduction after 20 L of processed plasma was 243 mg (204–262 mg; 95% CI 211–261 mg), which equals a reduction of 82.1% (77.0%–83.6%. 95% CI 78.8%–84.0%). The percentage diclofenac reduction decreased from 39.9% (25.3%–46.6%) at 0.2 L to 6.4% (−3.8–14.8) at 20 L of processed plasma.

**FIGURE 3:**
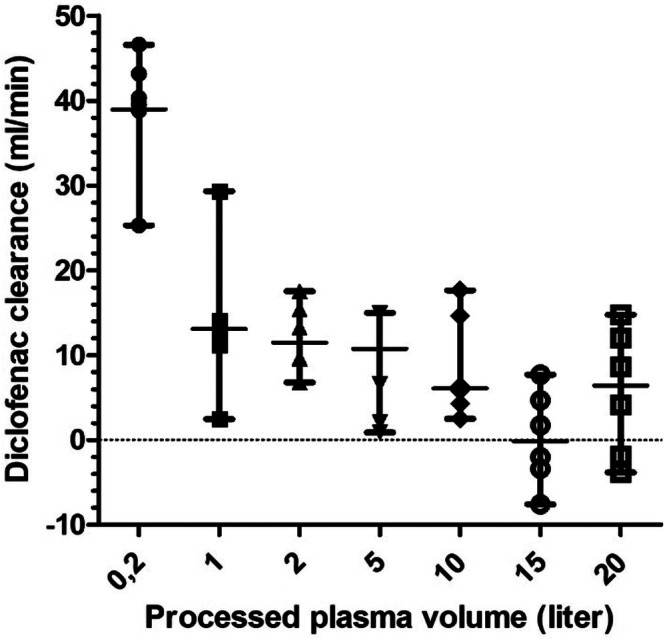
Median and range (minimum–maximum) clearance of diclofenac in porcine plasma during hemoadsorption using the CytoSorb adsorber at a plasma flow rate of 100 mL/min within six test runs. Median diclofenac clearance (mL/min) at different time points is represented by a circle, square, or arrowhead for every single test run. Overall median diclofenac clearance for all test runs is represented by the middle bar; overall minimum and maximum clearance is reported as lower and upper bar, respectively.

Total diclofenac clearance was 821 mL/200 min (770–836 mL/200 min; 95% CI 788–840 mL/200 min). The diclofenac clearance at a plasma flow of 100 mL/min decreased from 39.9 mL/min (25.3–46.6 mL/min) at 0.2 L of processed plasma to 6.4 mL/min (−3.8–14.8 mL/min) at 20 L of processed plasma (Figure 3).

### 3.2 Meloxicam

**FIGURE 4:**
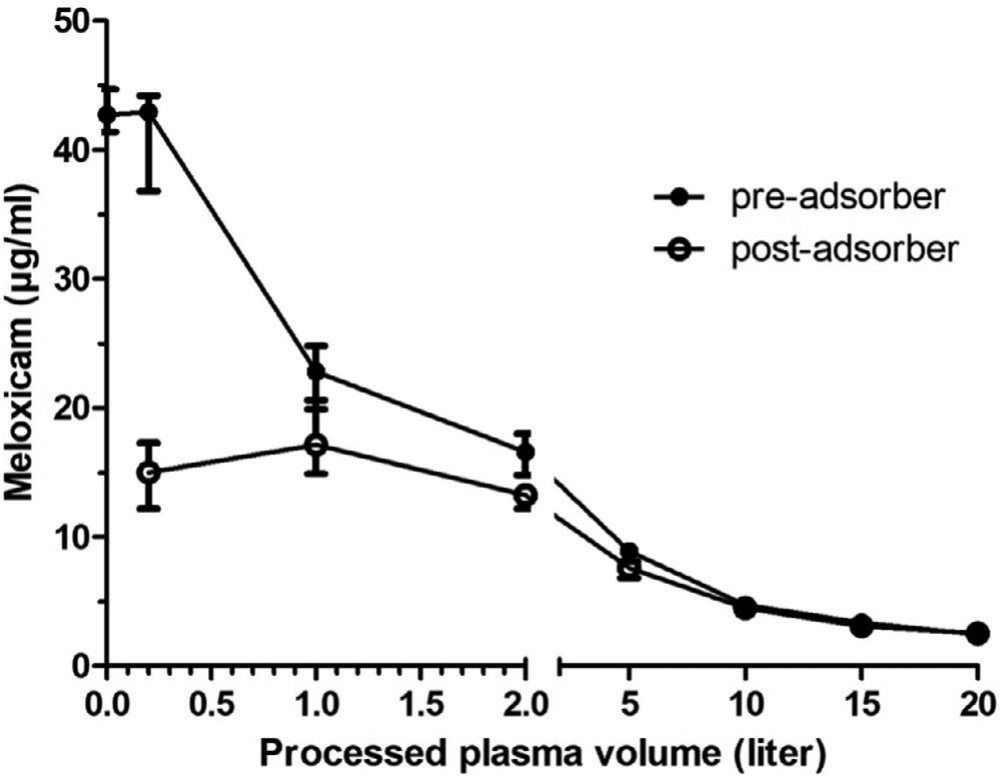
Median and range (min–max) of pre‐ and post‐adsorber concentrations of diclofenac in 1 L of porcine plasma spiked with 48 mg diclofenac during six test runs. Minimum and maximum concentrations (μg/mL) are represented by the lower and upper bar, respectively; median concentration is represented by a dot (pre‐adsorber) and a circle (post‐adsorber). Hemoadsorption was performed at a plasma flow rate of 100 mL/min using the CytoSorb adsorber.

Meloxicam had a recovery rate in porcine plasma of 99.5% with an intended concentration of 43 μg/mL, reaching a starting concentration of 42.8 μg/mL (41.4–44.7 μg/mL). The pre‐adsorber meloxicam concentration of 42.8 μg/mL (41.4–44.7 μg/mL) decreased significantly between the time points 0 and 10, 15 and 20 L, as well as between 0.2 and 10, 15, 20 L and between 1 and 20 L (*p* < 0.001, Figure 3, Table 2). The post‐adsorber meloxicam concentration of 15.0 μg/mL (12.2–17.3 μg/mL) at 0.2 L decreased significantly between the time points 0.2 and 15 and 20 L, as well as between 1 L and 10, 15 and 20 L, and between 2 L and 20 L (*p* < 0.001, Figure 4, Table 2).

**TABLE 2:** Meloxicam concentration in 1 L of porcine plasma spiked with 42.8 mg meloxicam during hemoadsorption using the CytoSorb adsorber.

Processed plasma volume (L)	*n*	0	0.2	1	2	5	10	15	20	*p* (Friedman test)
Meloxicam pre‐adsorber (μg/mL)	6	42.8 (41.4–44.7)	43.0 (36.8–44.2)	22.8 (19.9–24.8)	16.6 (14.8–18.0)	8.9 (8.1–9.1)	4.8 (4.6–5.1)^a^, ^b^	3.4 (3.1–3.6)^a^, ^b^	2.5 (2.3–2.8)^a^, ^b^, ^c^	**< 0.001**
Meloxicam post‐adsorber (μg/mL)	6		15.0 (12.2–17.3)	17.2 (14.9–20.6)	13.3 (12.2–13.7)	7.7 (6.8–8.2)	4.5 (4.3–4.7)^c^	3.1 (3.0–3.4)^b^, ^c^	2.6 (2.3–2.7)^b^, ^c^, ^d^	**< 0.001**
*p* (Wilcoxon matched pairs signed rank test)	6		**0.031**	0.063	**0.031**	**0.031**	**0.036**	**0.034**	0.583	

*Note:* Samples were collected before (pre) and after (post) the adsorber after processing 0–20 L of plasma. Concentrations are reported as median and range (minimum—maximum). Meloxicam values at each sample collection point (pre‐ adsorber and post‐adsorber) are analyzed using the Friedman test and post hoc Dunn's multiple comparison test. Concentration differences before and after the adsorber were analyzed using the Wilcoxon matched‐pairs signed‐rank test. The bold values indicate statistical significance based on *p* < 0.05.

Abbreviation: *n* = number of test runs.

^a^ Different to 0.

^b^ Differnt to 0.2 L.

^c^ Different to 1 L.

^d^ Different to 2 L.

The total meloxicam reduction after 20 L of processed plasma was 40.4 mg (38.6–42.2 mg; 95% CI 39.2–41.8 mg), which equals a decrease by 94.4% (93.2%–94.6%; 95% CI 93.6%–94.7%). The meloxicam reduction decreased from 65.4% (56.5%–71.7%) at 0.2 L to 3.6% (−13.0% to 4.2%) at 20 L of processed plasma.

**FIGURE 5:**
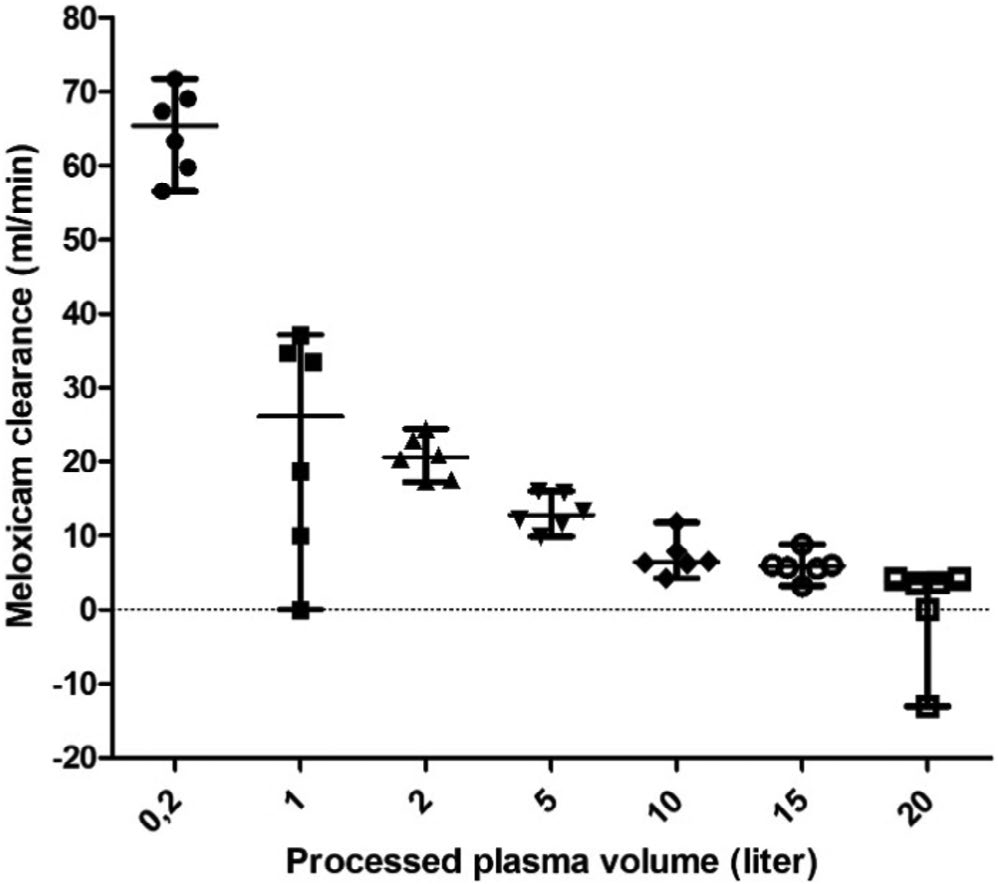
Median and range (minimum–maximum) clearance of meloxicam in porcine plasma during hemoadsorption using the CytoSorb adsorber at a plasma flow rate of 100 mL/min within six test runs. Median meloxicam clearance (mL/min) at different time points is represented by a circle, square or arrowhead for every single test run. Overall median meloxicam clearance for all test runs is represented by the middle bar; overall minimum and maximum clearance is reported as lower and upper bar, respectively.

Total meloxicam clearance was 944 mL/200 min (932–946 mL/200 min; 95% CI 936–947 mL/200 min). The meloxicam clearance at a plasma flow of 100 mL/min decreased from 65.3 mL/min (56.7–71.7 mL/min) at 0.2 L to 3.6 mL/min (−13.0–4.2 mL/min) at 20 L of processed plasma (Figure 5).

## 4 Discussion

In this study, the performance of the CytoSorb HA adsorber for in vitro NSAID removal was evaluated. The results of this study showed a reduction of diclofenac and meloxicam of 82.4% and 94.4% when compared to initial concentrations in porcine plasma treated with HA using the CytoSorb adsorber. Thus, in the present in vitro study, a considerable amount of substance removal from porcine plasma was achieved with HA using the CytoSorb adsorber for meloxicam and diclofenac in supratherapeutic doses. The majority of substance adsorption occurred in the first hour of treatment with HA, but clearance rates declined over time. Similar findings were previously reported in 2021 by Schneider et al. in an experimental in vivo study on the pharmacokinetics of selected anti‐infective drugs, including beta‐lactams, antifungals, and other anti‐infectives. In this study, the highest clearance was observed in the first 15 min of therapy for vancomycin, gentamicin, and teicoplanin [24]. Similarly, an in vitro study published in 2019 by König et al. found that the maximum substance adsorption for a panel of anti‐infectives in therapeutic concentrations can be seen within the first 60 min of treatment with CytoSorb adsorber in different carrier solutions (isotonic saline, human albumin solution and reconstituted blood) [23]. These observations align with the findings of the current study. However, to circumvent a possible influence on adsorber capacity due to concurrent analysis of multiple substances within a single carrier solution in this study, only one substance was added into a fresh unit of porcine plasma in each iteration.

An increasing number of case reports on the use of HA in clinical intoxications describe HA as a promising tool in the treatment of acute intoxications. Although direct comparison of these cases is challenging due to the heterogeneity of clinical cases and differences in timing of adsorber implementation and changes and concurrent therapeutic measures, these reports suggest highly efficient drug removal with the CytoSorb adsorber, in accordance with the current study [25, 26, 27, 28, 29].

A further finding in this in vitro study was the comparatively low spike recovery rate for diclofenac. The spike recovery rate of meloxicam in porcine plasma was 99.53%, whereas diclofenac showed a 68.46% recovery. To exclude the influence of the use of porcine, instead of human plasma on the results of the study, spike‐and‐recovery experiments were conducted for both substances, which yielded similar results regarding the recovery rate of diclofenac from porcine and human plasma. Thus, the difference in recovery rates for the two compounds may be related to distinct adsorption properties to plastic surfaces commonly used in medical supplies, such as syringes, intravenous fluid bottles, and tubing. To the authors' knowledge, no studies have directly quantified the adsorption of meloxicam to plastic. Hawkins et al. reported that meloxicam solutions retained over 90% of the original concentration after dilution and storage for 28 days at various temperatures. However, these solutions were stored in amber glass prescription bottles rather than plastic [31]. In contrast, diclofenac has been demonstrated to adsorb to plastic surfaces, which may contribute to its considerably lower recovery compared to meloxicam [32, 33, 34]. Another possible explanation for the observed compound‐specific difference in recovery lies in the composition of porcine plasma and drug–protein binding. Talaj et al. investigated structural differences in serum albumins from ovine, caprine, and leporine species compared to human and equine albumins and found that these differences significantly affected the free diclofenac concentration. Notably, the albumin of these species possesses two common and multiple unique binding sites for diclofenac, none of which overlap with the binding sites found in human albumin [35]. These findings suggest that porcine plasma may similarly contain distinct diclofenac binding sites, potentially influencing drug recovery. To the authors' knowledge, no studies have specifically quantified diclofenac recovery from porcine plasma in vitro.

In the present study, the percentage reduction of diclofenac decreased from 39.9% at 0.2 L of processed plasma to 6.4% after 20 L. Similarly, the percentage reduction of meloxicam decreased from 65.4% at 0.2 L to 3.6% at 20 L of processed plasma. This decrease in percentage reduction is likely caused by the concentration‐dependent operating principle of the adsorber, leading to high reduction rates at high substance concentrations and decreasing reduction quantities at lower concentrations, as well as progression of adsorber saturation.

For this experiment, it was hypothesized that meloxicam and diclofenac would be well adsorbed during HA due to their physical and chemical properties. Both substances are highly protein‐bound, low MW, hydrophobic compounds, with a VD of 0.3 and 0.1–0.2 L/kg, respectively, thus providing excellent properties for adsorption by the CytoSorb adsorber [5, 9]. Optimal physical and chemical properties of a substance to be effectively removed by HA are a MW < 40 kDa, low or high protein binding, a VD < 1 L/kg and water or lipid solubility. In in vivo settings, the endogenous clearance rate of the respective substance should be taken into account when choosing an extracorporeal treatment modality [15]. The current study showed an effective reduction of more than 80% from baseline concentrations after processing 20 plasma volumes, which represents a circulation time of 200 min for both drugs assessed. These findings suggest that HA using the CytoSorb adsorber can be a suitable treatment option for poisonings with high doses of meloxicam or diclofenac. Further studies are needed to elucidate the translatability of the results to in vivo settings.

The high reduction capability observed in this study is most probably a combined result of the CytoSorb adsorber's high surface‐to‐volume ratio, both substances' physical and chemical properties, plasma flow, and the concentration‐dependent modus operandi of HA. In the CytoSorb adsorber used in this study, the sorbent material consists of highly porous coated sorbent polymer beads in a cartridge, providing an extensive adsorbent surface area of approximately 45 000 m^2^ [13]. Solute binding in the CytoSorb adsorber occurs in three steps. First, plasma flow is directed into the interparticle space in the cartridge. Second, external mass transfer of the solute from the plasma occurs by convection to the outer sorbent surface. The last step to adsorption is called internal mass transfer and refers to the pore diffusion of the substance to the inner surface of the internal porous structure of the sorbent bead [30]. An optimal plasma flow rate for one‐compartment in vitro experiments investigating drug removal capacity of the CytoSorb adsorber from plasma has not yet been established. A plasma flow of 100 mL/min was chosen for this experiment, as to allow for sufficient contact time between the sorbent bead surface and the plasma for maximum solute binding. For optimal clinical results whilst at the same time reducing the risk of adsorber clotting, higher flow rates of 150–350 mL/min have been reported and recommended by the manufacturer [16, 17, 18, 24, 36]. These flow rates are only evaluated for clinical purposes and might be different for optimal drug removal in an experimental setting. Other in vitro experimental studies, on the other hand, also used flow rates below 100 mL/min. However, it must be noted that the experiment was conducted using a down‐scaled version of an extracorporeal circulation system with smaller adsorber cartridges in one case and with the use of a cell‐free mixture of 0.9% NaCl and human albumin 5% in the other, necessitating adjustments in flow rates [23, 37].

The findings of the present study show approximation of pre‐ and post‐adsorber concentrations at 10 and 15 L of processed plasma, equaling a circulation time of 1.6 and 2.5 h for meloxicam and diclofenac, respectively. This means that there is an equilibrium between adsorption and desorption of the substance at that time point. This equilibrium can be explained in two ways. Due to the single‐bolus and one‐compartment model of this setup, there might be a dynamic equilibrium because of the reduced concentration gradient between pre‐ and post‐adsorber. Another explanation would be an absolute saturation of the adsorber because the binding capacity of the cartridge is fully exhausted. This would occur as a direct consequence of saturation of all available adsorber binding sites, impeding further substance adsorption.

The results of the current study show further, albeit slight, substance reduction, despite prior equilibration of pre‐ and post‐adsorber concentration. It cannot be excluded that some other physical or biochemical factors such as temperature, oxidation, or light exposure caused a further self‐degradation of the substance after the adsorption equilibrium was achieved.

As shown in the current study, HA is effective in reducing very high concentrations of diclofenac to a safe level for elimination via the patient's own metabolic pathways. Considering the reportedly strong safety profile of this device, CytoSorb can be considered a valid treatment option in acute and life‐threatening intoxications where a direct antidote is not available or for poisonings with anticipated development of toxic metabolites [13, 16]. Definitive conclusions regarding the in vivo translatability of the results of this in vitro study are difficult to draw. The performance of prospective clinical trials is necessary but remains challenging to accomplish. Clinical applicability and effectiveness greatly depend on various factors, such as patient characteristics, availability of extracorporeal treatment techniques, and substance characteristics. In vivo, HA would be a rational choice if the rate of elimination with HA is deemed to be expectedly faster than the elimination half‐life of the substance. As diclofenac has a considerably shorter elimination half‐life than meloxicam (1–2 and 20 h, respectively) [6], the clinical usefulness of HA in diclofenac overdose in vivo is debatable. However, with ingestion or intravenous administration of extremely high doses or concurrent impairment of renal or hepatic function, HA might still be a useful addition to symptomatic treatment. Furthermore, saturation of metabolic elimination pathways due to high drug concentrations or illness‐related impairment of excretory function, as in organ failure, might prolong elimination time, putting the patient at risk for substantial or further organ damage due to drug metabolism.

### 4.1 Limitations

This study has some limitations, which are attributable to the in vitro experimental design and set‐up characteristics. Although the use of plasma instead of whole blood does not exactly mirror the clinical situation, the results are not expected to differ from the use of whole blood, as the CytoSorb adsorber demonstrates excellent hemocompatibility with minimal influence on cellular blood component number and function [13, 16, 38]. To account for the higher effective plasma flow in this one‐compartment model using plasma instead of whole blood, which could theoretically result in a higher adsorption per unit of time, measured results were evaluated under consideration of the plasma flow per time unit.

An impact of substrate temperature on toxin removal capacity of the CytoSorb adsorber cannot be excluded. As all adsorption processes are temperature dependent, a physiologic temperature of 37°C might have changed the results in this experimental setup. Higher temperature might have caused an altered interaction of proteins with the adsorber surface, altered plasma viscosity, or an altered fraction of non‐protein‐bound substance. Therefore, the results of this study cannot be transferred to a setup with a standardized temperature of 37°C.

A further limitation is a given scaling mismatch between the plasma volume, size of the adsorber cartridge and total plasma substance load with possible influence on results, warranting cautious interpretation regarding their transferability to clinical practice. For study purposes, the plasma volume of each unit was reduced to 1 L, as opposed to 3.5 L plasma volume in a normal 70 kg adult, and each unit was spiked with an equivalent dose of the respective drug for the reduced plasma volume. Consequently, the absolute drug amounts applied in our experiments were lower than those expected in clinical adult overdose scenarios, resulting in a relative underloading of the adsorber. This might lead to an overestimation of substance reduction capacity and delay in adsorber saturation as compared to its use under clinical conditions. Due to the unavailability of smaller adsorbers, the experiment was carried out using a normal‐sized adsorber cartridge for this down‐scaled one‐compartment model. While this experimental set‐up might to some extent influence adsorption dynamics, the study still provides evidence for the capability of HA using the CytoSorb adsorber to remove meloxicam and diclofenac from porcine plasma in vitro.

Another limitation harbored in the one‐compartment model study design is that in vivo pharmacokinetics, such as the rebound effect due to redistribution between the interstitial and intravascular compartments after treatment discontinuation, cannot be evaluated. With a VD of 0.3 L/kg, meloxicam is readily distributed into the interstitium, causing an expected rebound after treatment with HA. This rebound effect would negatively influence the total reduction rate in vivo. This is not as pronounced with diclofenac, with a VD of 0.1–0.2 L/kg, rendering it more accessible for extracorporeal removal. Contrarily, in an in vivo model, alternative routes of clearance and elimination work simultaneously, aiding in the clearance of those substances, further reducing the toxicant concentration. The rebound effect can be mitigated by considerate timing of adsorber changes, if saturation is suspected or a measured equilibrium pre‐ and post‐adsorber is reached [30]. Furthermore, effective reduction of rebound effects can be reached by prolonged duration of HA, as most adsorbers can be operated continuously for 24 h without the need to pause the procedure.

## 5 Conclusion

HA using the CytoSorb adsorber shows promising results to rapidly decrease toxic concentrations of meloxicam and diclofenac in vitro. Further in vivo studies are needed to evaluate the clinical usefulness of HA with the CytoSorb adsorber in acutely and severely intoxicated patients in practice and its applicability in the treatment of acute NSAID overdose.

## Conflicts of Interest

The authors declare no conflicts of interest.

## Data Availability

The data that support the findings of this study are available on request from the corresponding author. The data are not publicly available due to privacy or ethical restrictions.
